# Surfactant-Assisted Fabrication of Alumina-Doped Amorphous Silica Nanofiltration Membranes with Enhanced Water Purification Performances

**DOI:** 10.3390/nano9101368

**Published:** 2019-09-24

**Authors:** Xianzheng Ma, Katarzyna Janowska, Vittorio Boffa, Debora Fabbri, Giuliana Magnacca, Paola Calza, Yuanzheng Yue

**Affiliations:** 1 Department of Chemistry and Bioscience, Aalborg University, Fredrik Bajers vej 7H, 9220 Aalborg, Denmark; xm@bio.aau.dk (X.M.); kaj@bio.aau.dk (K.J.); yy@bio.aau.dk (Y.Y.); 2 Department of Chemistry, University of Turin, Via P. Giuria 5/7, 10125 Torino, Italy; debora.fabbri@unito.it (D.F.); giuliana.magnacca@unito.it (G.M.); paola.calza@unito.it (P.C.); 3 NIS Interdepartmental Center, Universitá di Torino, Via P. Giuria 7, 10125 Torino, Italy

**Keywords:** sol-gel, wastewater, depollution, desalination, selectivity

## Abstract

Surfactant-templated 5 mol% Al_2_O_3_-doped silica membranes nanofiltration membranes were synthesized via the sol-gel method, and afterward, were optimized, and tested with respect to the permeability and rejection rate. The disordered silica network was stabilized by doping 5 mol% alumina. Tetraethyl orthosilicate and aluminum isopropoxide were used as the silica and alumina precursors, respectively. Cetyltrimethylammonium bromide (CTAB) was used not only as a pore-forming agent, but also to control the reaction rate of the aluminum isopropoxide, thus obtaining highly homogeneous materials. The results about filtration of model solutions showed that the optimized membranes are featured by both a relatively high water permeability (1.1–2.3 L·m^−2^·h^−1^ ·bar^−1^) and a high rejection for salts (74% for NaCl, and >95% for MgSO_4_ and Na_2_SO_4_) and organic pollutants (e.g., about 98% for caffeine). High rejection of divalent ions and organic molecules was also observed when a real wastewater effluent was filtered. The influence of the synthesis conditions on the membrane performance is discussed.

## 1. Introduction

Water scarcity is one of the most pressing challenges for the human population. Moreover, the growing demand for clean water and the limited access to water resources for a large part of the global population urgently require sustainable approaches to address this problem, without compromising water access for the future generations [1,2]. Various approaches have been developed to address water depollution in an energy-efficient manner, including adsorption, filtration, electrochemistry, and photocatalysis [3]. In this context, pressure-driven membrane filtration systems, such as nanofiltration (NF), have become increasingly popular since they do not require any chemical treatment or thermal input to the treated water. NF membranes have a pore size ranging between 1 and 2 nm. Therefore, such membranes are efficient for removing multivalent salt ions and small organic pollutants [2,4,5]. Hence, NF membranes might represent one of the possible solutions to the issues of water scarcity and water pollution. Currently, polymeric membranes dominate the NF market, because they can offer a good compromise between selectivity and water permeability. Indeed, commercial polyamide NF membranes can achieve a rejection of about 97% for divalent ion salts (MgSO_4_) while maintaining a good permeability (i.e., 6.7–10.9 L·m^−2^·h^−1^·bar^−1^) [6,7,8]. However, polymer membranes have a low tolerance for harsh mechanical and chemical conditions. Membrane fouling requires frequent chemical cleaning, which limits their usage and lifespan [9,10]. On the other hand, inorganic NF membranes show great potential for water desalination and detoxification, because they are easy to be cleaned and have a long service life [11,12]. Nevertheless, inorganic NF modules have low water permeability and filtering area density compared to the polymer membranes, preventing them from practical application in water filtration [13].

Surfactant-templated amorphous (i.e., non-crystalline) silica represents ideal membrane materials, due to their high pore volume and narrow pore size distribution in a range suitable for NF. Surfactants are applied as sacrificial templates to create nanopores during material consolidation and calcination, thus tailoring the porosity of the final membrane material [14]. However, amorphous silica (*a*-silica) membranes are unstable in basic solutions [15], and in the hydrothermal environment [16,17]. Hence, this limits the perm/selectivity and the field of usage of *a*-silica membranes. The stability of the *a*-silica framework can be enhanced by doping with metal oxides. Moreover, doping can alter interfacial properties, morphology, hydrophilicity, and surface charge of the membranes [13,14,18], and therefore, improve membrane performances, such as ion rejection and fouling resistance [3,18].

The stability of several metal oxide–silica compositions, such as cobalt oxide–silica, zirconium oxide–silica, and titanium oxide–silica, etc., have been studied [15,19,20]. In particular, the enhanced stability has been observed for the SiO_2_-Al_2_O_3_ film-based membrane [21]. The Al_2_O_3_-SiO_2_ system is often used for the fabrication of zeolite membranes, which are highly stable and can achieve high rejection for ions and micropollutants. However, their crystalline structure makes it difficult to reduce the thickness of the membrane layer to below 1 µm, thus limiting their permeability, which is usually lower than 0.1 L·m^−2^·h^−1^·bar^−1^ [22]. On the other hand, surfactant-templated *a*-silica consists of a long-range disordered network with an open structure, which can be synthesized via a simple sol-gel method and deposited as films with the thickness of a few hundred nanometers by dip-coating. Moreover, surfactant-templated silica possesses pores with size ranging from 1.5 to 10 nm, while zeolites have typically pore size of <1 nm. Therefore, surfactant-template silica membranes typically allow for much greater water fluxes than zeolite membranes [23]. 

When synthesizing the binary silica-alumina films for membranes by the sol-gel method, it might be a challenge to obtain homogeneous colloids, since aluminum alkoxides have a much higher hydrolysis rate than tetraethyl orthosilicate (TEOS) [24]. A two-steps synthesis approach is often applied to obtain homogeneous sol systems when two precursors have different reactivities [13,25,26]. The first step is the pre-hydrolysis of the precursor with the lowest reactivity. The second step is the addition of the precursor with the highest reactivity. Previous studies have shown that surfactant molecules can also reduce the hydrolysis and condensation rate of alkoxide precursors, as they can interact with the metal center in the forming particles and limiting the reactions rates [25,26]. Hence, the surfactant was introduced into the reaction mixture after the pre-hydrolysis of TEOS, but before adding the aluminum alkoxide. Therefore, the surfactant had a dual function: (i) To control the hydrolysis and the condensation rate of precursors during synthesis, and (ii) to control the pore structure, as sacrificial pore-forming agent during material consolidation and calcination. Indeed, precipitation of Al_2_O_3_ from the sol was always observed when the surfactant was added after the second step. Nevertheless, depending on the synthesis conditions and sol composition, phase separation might occur in the final membrane material when the molar ratio of the alumina reach to 10% [27,28,29].

In this work, the 5 mol% Al_2_O_3_-doped silica membranes NF membranes were fabricated by optimizing the concentration of the coating sol and the molar ratio of surfactant/oxide (S/O). The impact of these fabrication parameters on membrane selectivity and permeability was investigated by performing filtration tests with model solutions. Then, the optimized membranes were tested regarding their ability to retain ions and pollutants in a real wastewater sample. 

## 2. Experimental

### 2.1. Sol-Synthesis and Membrane Fabrication

**Figure 1:**
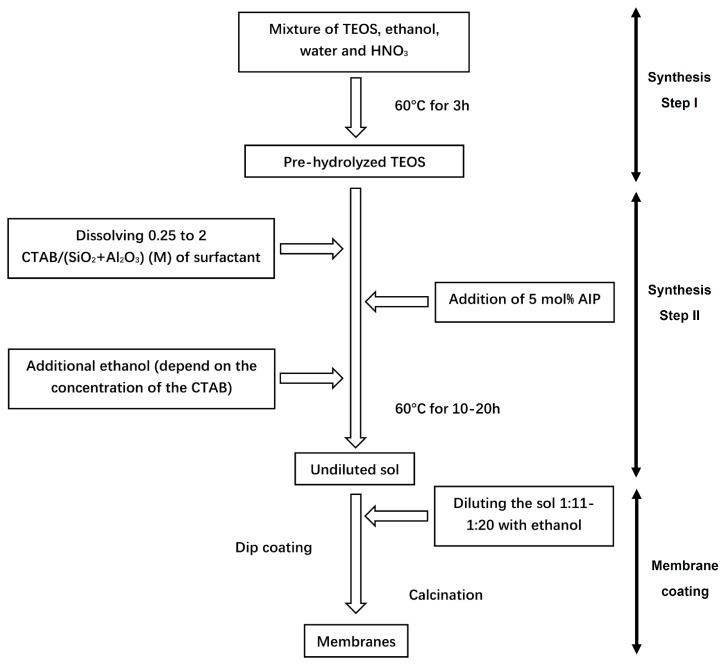
Synthesis process of the membranes with different cetyltrimethylammonium bromide (CTAB) concentration.

The fabrication procedure of our 5 mol% Al_2_O_3_-doped silica membranes is depicted in Figure 1. A two-steps approach was applied for the sol synthesis. The first step of synthesis was the hydrolysis of TEOS, which was achieved by letting to react a mixture of TEOS (98%, Sigma Aldrich, St. Louis, MO, USA), ethanol (99.9%, VWR Chemicals, Radnor, PA, USA), distilled water, and nitric acid (69%, Sigma Aldrich, St. Louis, MO), with a molar ratio of 1:4:2.5:0.04, at 60 °C for 3 h. Then, in the second step of the synthesis, CTAB (99%, Sigma Aldrich, St. Louis, MO, USA) was added to the pre-hydrolyzed TEOS solution to achieve the desired CTAB: (SiO_2_ + Al_2_O_3_) molar ratio. After the complete dissolution of CTAB, aluminum isopropoxide (AIP) (98%, Sigma Aldrich, St. Louis, MO, USA) was directly added to the mixture to obtain a 5 mol% Al_2_O_3_ concentration in the final consolidated membrane material. The mixture was continuously stirred at 60 °C until all the AIP was dissolved and a transparent yellowish solution was obtained (the reaction times are summarized in Table 1). 

Sols were diluted by 1:11, 1:15, and 1:20 volume ratios with ethanol and subsequently filtered with 0.2 μm syringe filter (Minisart RC, 25 mm, Sigma Aldrich, St. Louis, MO, USA) to remove dust particles and impurities before the coating. The membranes were coated on commercial α-alumina tubular support with a γ-alumina intermedia layer (250 × 10 × 7 mm (L × OD × ID), Pervatech B.V., Rijssen, The Netherlands). The membranes were fabricated by dip-coating of the alumina-doped silica sols onto the supporting substrates. Specifically, the inside of the supports was coated vertical by a lab-made device at a dipping/withdrawing rate of <2.5 cm/min. After drying at room temperature for 24 h, the membranes were calcined at 450 °C for 2 h at the heating and cooling rate of 2 °C/min.

The corresponding powdered samples were obtained by filtering the rest of coating sol through a 0.2 μm syringe filter after dilution with ethanol (1:1 V/V) and dried in Petri-dishes. The membrane materials were calcined by following the same temperature ramp of the supported membranes. After the calcination, the membrane materials were crushed in a mortar and kept for further analysis.

### 2.2. Membrane Characterization

The pore structure of the membrane materials was investigated by nitrogen adsorption at liquid-nitrogen boiling point on a gas volumetric apparatus ASAP 2020 (Micromeritics, Norcross, GA, USA), after outgassing at 300 °C in the vacuum (residual pressure 10^−2^ mbar) to avoid undesired interferences of gaseous products from materials during the gas-volumetric determinations. Specific surface areas were determined using the Langmuir model. Pore volumes and pore size distributions were calculated by using the density functional theory (DFT) method to examine simultaneously both micro and mesoporosity of the samples [30]. The morphology of the membrane cross-section and surface was investigated by SEM analysis using a EVO 50 XVP microscope (Zeiss, Köln, Germany) with LaB_6_ source. The samples were mounted on metallic stubs with double-sided conductive tape and ion coated with a gold layer (thickness ~25 nm) by a sputter coater (Baltec SCD 050, Pfäffikon, Switzerland) for 60 s under vacuum at a current intensity of 60 mA to avoid any charging effect.

### 2.3. Filtration Tests

The cross-flow setup for the filtration experiment is described in detail elsewhere [31]. Water permeability was measured under four different operating pressure ranges from 5 to 7 bar. The following reagents were dissolved in deionized water to prepare model solutions for the filtration test: NaCl (99.0%, Chemsolute, Roskilde, Denmark), MgSO_4_∙7H_2_O (99.0%, Acros Organics, Geel, Belgium), Na_2_SO_4_, (99.0%, Sigma Aldrich, St. Louis, MO, USA), and caffeine (Sigma Aldrich, St. Louis, MO, USA). Salt solutions with an ionic strength of 0.01 M and a caffeine solution (10 ppm) were prepared as feed for selectivity measurements. The selectivity tests were operated at 7 bar, the salt rejection was determined by measuring the electrical conductivity of the feeding and permeate water by using a conductivity meter Seven Multi (Mettler Toledo, Columbus, OH, USA). Caffeine concentration was determined by HPLC over a Dionex ASI-100 chromatograph with a Phenomenex Luna C18 column, with diameter, length, and pore size of 4.60 mm, 250 mm, and 5 μm, respectively. The mobile phase was deionized water (buffered with 0.025 M KH_2_PO_4_) and acetonitrile (ACN) with a proportion ACN/buffer of 20/80, delivered at a flow rate of 1.2 mL·min^−1^.

In order to test the performance of membranes towards the inorganic ions and organic pollutants, we analyzed the wastewater effluent and the permeate for the presence of some ions (Cl^−^, NO_3_^−^, SO_4_^2−^, Na^+^, K^+^, Mg^2+^, Ca^2+^) and for the presence of contaminants of emerging concern (in particular imidacloprid (IMI), ciprofloxacin (CPX), carbamazepine (CBZ), 1,2,3-benzotriazole (BZT) 5-methyl-1H-benzotriazole (MBZT). The content of total organic carbon (TOC) and total carbon (TC) was evaluated before and after the filtration step. The concentration of these target parameters was determined according to the procedures, which are described in detail in the Supplementary Materials (MS-MS conditions are reported in Table S1).

## 3. Results and Discussion

### 3.1. Impact of CTAB Concentration on Membrane Porosity

**Figure 2:**
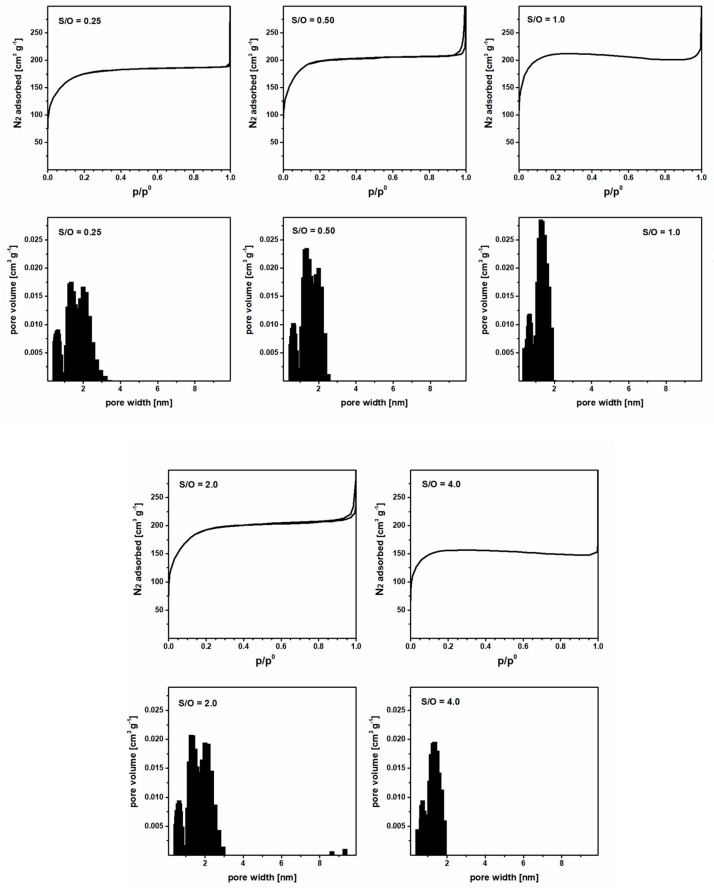
Adsorption isotherms and pore size distributions of the membrane materials (5 mol% Al_2_O_3_–doped silica) synthesized by the sol-gel method with different surfactant/oxide molar ratio (S/O) in the reaction mixture.

**Figure 3:**
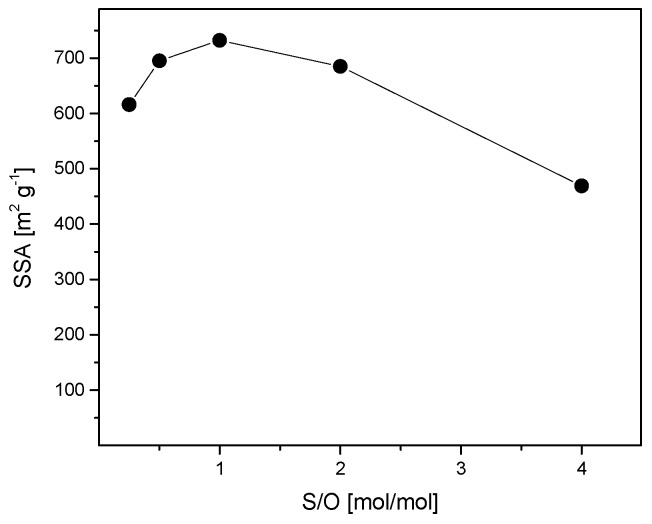
Specific surface area (SSA) of the membrane materials (5% Al_2_O_3_–doped *a*-silica) as a function of the surfactant/oxide molar ratio (S/O) in the reaction mixture.

The pore structure of the membrane materials with five different CTAB concentrations (S/O = 0.25, 0.5, 1.0, 2.0, and 4.0) was determined by low-temperature N_2_ adsorption. Despite the different CTAB concentrations, all the samples have similar adsorption isotherms and pore size distributions, as shown in Figure 2. Most of the adsorption occurs at a nitrogen relative pressure smaller than 0.1 (type I isotherms, according to the IUPAC classification, which is typical of microporous materials [32]). As a consequence, the pore size of the membrane materials ranges from 0.5 to 2.5 nm. This pore size distribution is consistent with the use of CTAB as the pore-forming agent. The relation between the CTAB concentration and specific surface area (SSA) of the membrane materials is plotted in Figure 3. At first, SSA increases with increasing CTAB concentration. SSA reaches a maximum at 732 m^2^·g^−1^ when the surfactant/oxide molar ratio (S/O = CTAB/(SiO_2_ + Al_2_O_3_)) is 1. Any further increase of CTAB concentration results in a reduction of the specific surface area, which may be caused by the collapse of the pore structure when a high amount of surfactant is burned out during calcination.

### 3.2. Concentration of the Coating Sol

**Figure 4:**
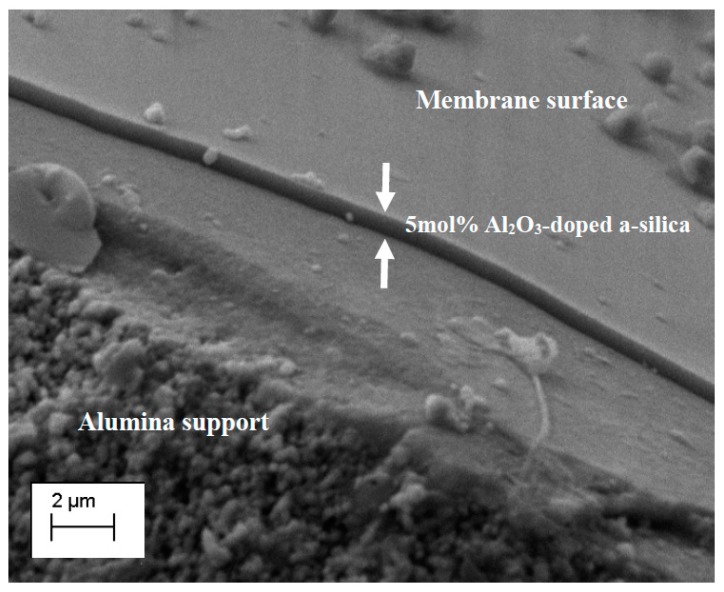
Cross-sectional SEM micrograph of the membrane obtained by coating a 5 mol% Al_2_O_3_-doped silica sol with a surfactant/oxide ration (S/O) of 0.25 and an oxide (SiO_2_ + Al_2_O_3_) concentration of 11.8 g·L^−1^. White arrows indicate the 5 mol% Al_2_O_3_-doped silica layer.

The membrane materials were deposited on commercial membrane tubes by dip-coating. Different dilutions were used and Table 1 summarizes the oxide (SiO_2_ + Al_2_O_3_) concentrations of the final coating sols. Figure 4 shows the SEM image of a membrane obtained from a sol with S/O = 0.25 and oxides (SiO_2_ + Al_2_O_3_) concentration of 11.8 g·L^−1^. The micrograph shows a continuous membrane layer covering the multi-layered alumina support. Form the picture, the thickness of the membrane is estimated to be around about 590 nm. Therefore, we attempted to reduce the membrane thickness and increase membrane permeability by dilution of the coating sol. Two new membranes were prepared by reducing the oxide concentration in the sol from 11.8 to 8.7 and 6.5 g·L^−1^ while S/O was kept constant to 0.25.

**Figure 5:**
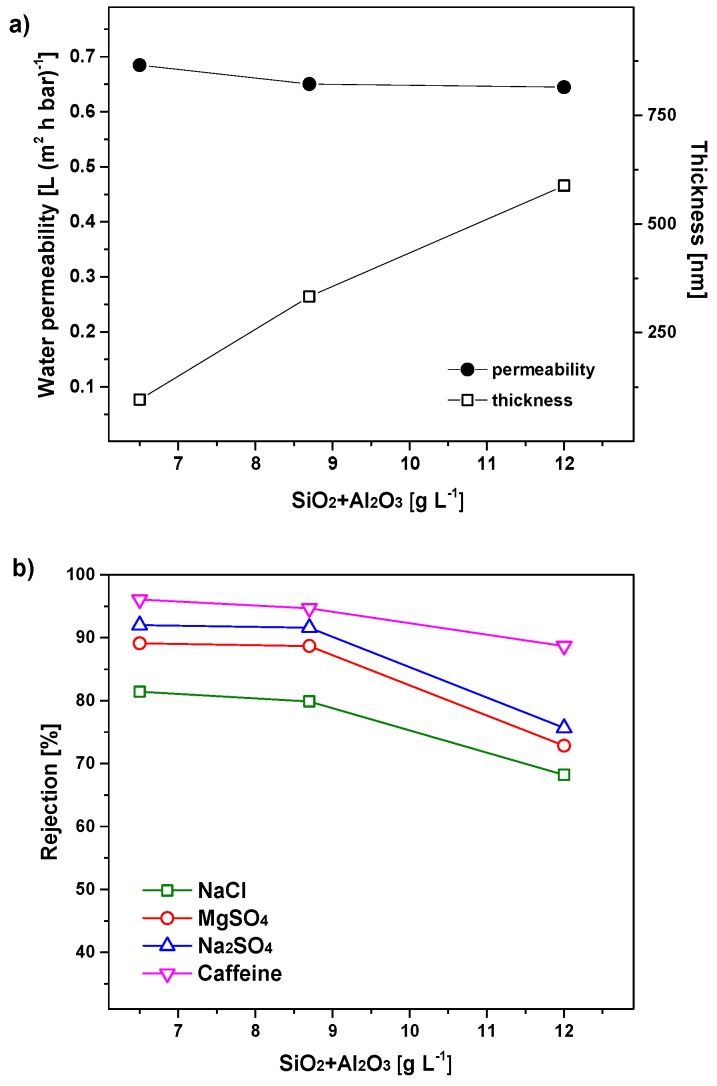
(**a**) Water permeability and thickness of 5 mol% Al_2_O_3_-doped silica membranes as a function of the SiO_2_ + Al_2_O_3_ concentration in the coating sol. (**b**) Membrane rejection for inorganic salts and caffeine as a function of the SiO_2_ + Al_2_O_3_ concentration in the coating sol.

SEM images of the membrane cross-section were taken to compare the thickness of the membranes with different sol concentrations. The relation between the membrane thickness and the sol dilution is reported in Figure 5a. The thickness of the 5 mol% Al_2_O_3_-doped silica membrane layer decreases by reducing the oxide concentration in the coating sol: From 560 nm to 96 nm when the sol oxide concentration was reduced from 11.8 to 6.5 g·L^−1^. Surprisingly, the difference in membrane thickness did not reflect in a large variation of the water permeability of the three membranes, as shown in Figure 5a. Indeed, when the oxide concentration in the sol decreases from 11.8 to 6.5 g·L^−1^ the average water permeability has only changed slightly: From 0.64 to 0.68 L·m^−2^·h^−1^·bar^−1^, that is, the change of the water permeability is negligible when compared to the decrease of the membrane thickness. This may be caused by the infiltration of the sol particles into the porous intermedia layer during the coating, resulting in a much thicker 5 mol% Al_2_O_3_-doped silica layer than that observed in the SEM images.

The rejection of the membranes to inorganic salts and caffeine (as a model micropollutant) is shown in Figure 5b. Rejection values of the three membranes are consistent with NF membrane layers. Indeed, the three membranes show good rejection for salts of monovalent ions (68–81% for NaCl) and high rejection towards salts of divalent ions (73–89% for MgSO_4_ and 76–92% for Na_2_SO_4_), and even higher rejection for caffeine (89–96%). In general, the membranes ionic rejection depends on the size and the charge of the hydrated ions. For non-charged species such as caffeine, the rejection mechanism is mainly attributed to the steric exclusion. These results indicate that the three membranes consist of NF layers with a really small or negligible defect density. Counterintuitively, the rejection of ions and caffeine have an increasing trend with the decrease of the membrane thickness. For instance, the rejection for NaCl is increased from 68.2% to 81.4% when the oxide concentration in the coating sol decreases from 11.8 to 6.5 g·L^−1^. A possible explanation could be that thicker films have a higher chance of defect formation during drying [33], as all the membranes have the same composition and have similar pore structure. This result shows that dilution of the coating sol could improve the selectivity without sacrificing the membrane permeability. Therefore, we decided to prepare the membranes by using the coating sol with a rather low oxides concentration (6.5 g·L^−1^) in the rest of the study.

### 3.3. Optimization of the S/O Ratio

**Figure 6:**
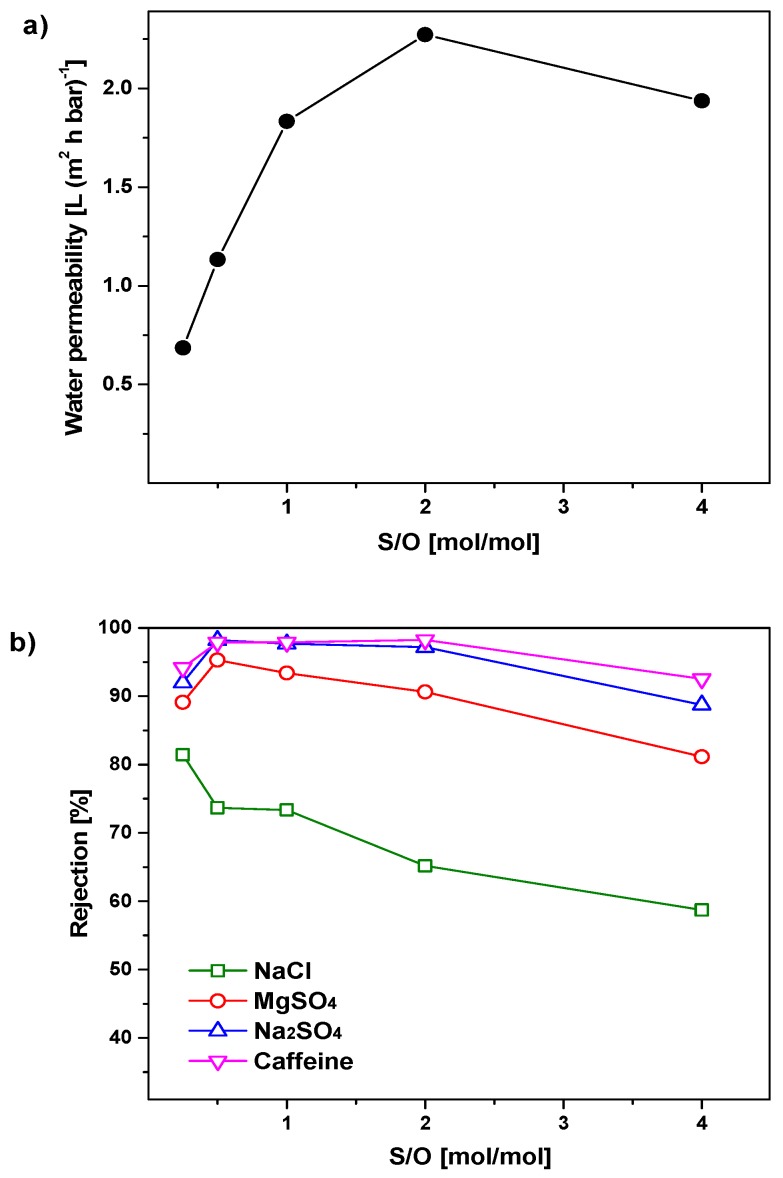
(**a**) Water permeability and (**b**) rejection to salt and caffeine of the 5 mol% Al_2_O_3_-doped silica membranes as a function of the surfactant/oxides (S/O) ratio in the coating sols.

Figure 6a illustrates the relationship between the water permeability of the membranes and the surfactant/oxides ratio (S/O = 0.25, 0.5, 1.0, 2.0, and 4.0). A dramatic increase of the water flux is observed when S/O is increased from 0.25 to 2.0. In this S/O range, the permeability increases rapidly from 0.68 to 2.3 L·m^−2^·h^−1^·bar^−1^. However, a further increase of the surfactant concentration (S/O from 2.0 to 4.0) results in a reduced permeability (1.9 L·m^−2^·h^−1^·bar^−1^). The initial rise of the permeability for increased S/O ratios may arise from increased pore interconnectivity. However, when the concentration of surfactant is fourfold higher than the oxide materials, pore walls might collapse during calcination. 

The rejections of these membranes for inorganic salts and caffeine are depicted in Figure 6b. The rejection for NaCl decreases from 81% to 59% when the surfactant concentration (S/O) increases from 0.25 to 4.0. On the contrary, the rejections for MgSO_4_, Na_2_SO_4,_ and caffeine reach a maximum (95%, 98%, and 98%, respectively) for the membrane prepared from a sol with S/O of 0.50. The increase of membrane rejection with increasing S/O from 0.25 to 0.50 could be due to the fact that surfactant molecules contribute to reduce capillary stresses during the drying of the membrane films, thereby reducing the probability of defect formation [34]. However, when further increasing the surfactant concentrations in the coating sols, the rejection decreases for the tested three types of ions and caffeine. This trend might be ascribed to the formation of some defects in the membrane films due to the high relative mass loss and gas product development during the calcination step. 

As expected, the effect of S/O on the membrane rejection is not as strong as what we observed for the membrane permeability. Indeed, as shown in Figure 2 and Figure 3, the surfactant concentration has little effect on the pore size distribution, and thus on the membrane selectivity. However, it has a great impact on the specific surface area of the material, and therefore, on the membrane permeability. In detail, the membrane with S/O = 2.0 has the highest permeability, while the membrane with S/O = 0.5 exhibits the best selectivity. These two membranes present selectivity close to those of zeolite membranes [35], but water permeability at least one order of magnitude higher. Therefore, they were selected as optimized membranes for filtering the effluent of the wastewater treatment plant.

### 3.4. Filtration of A Wastewater Treatment Plant Effluent

**Figure 7:**
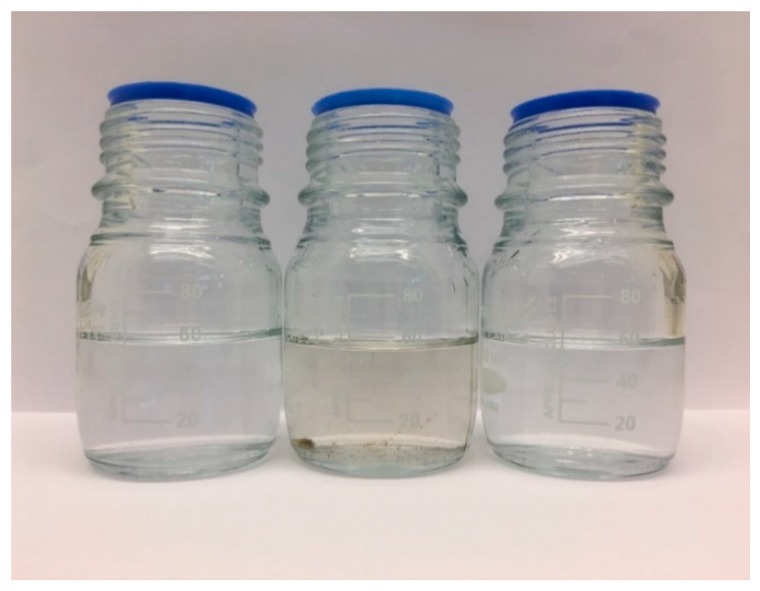
Samples of wastewater treatment plant effluent (**middle**), permeate of the membrane with surfactant/oxide ratio (S/O) = 0.5 (**left**), and permeate of the membrane with S/O = 2.0 (**right**).

Figure 7 shows the wastewater sample before filtration and the permeates collected after the wastewater was filtered through the membranes with S/O = 0.50 and 2.0. The effluent water presents a slightly yellowish color and some turbidity. On the contrary, the permeate water from both membranes is colorless and clear. This picture shows that the membranes have the ability to remove colloids and colored compounds (as humic substances) in the wastewater. As a result of the accumulation of these substances on the membrane surface, the permeability of the two membranes after 2 h of filtration was reduced to 0.16 and 0.32 L·m^−2^·h^−1^·bar^−1^ for the membranes with S/O = 0.50 and S/O = 2.0, respectively. 

**Figure 8:**
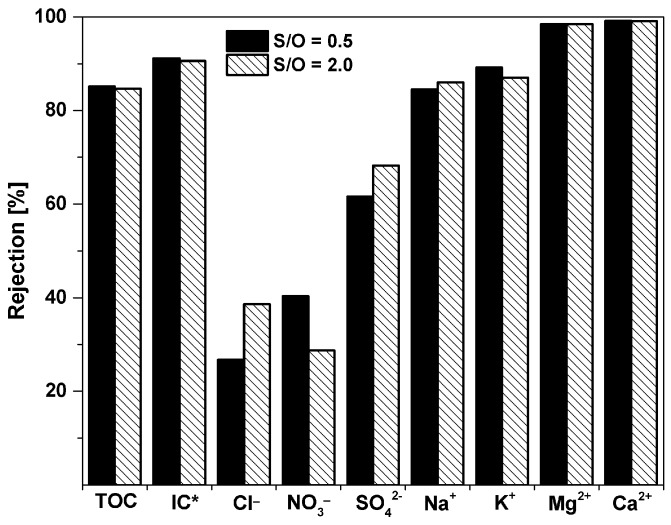
The rejection of the ions, micropollutants, and organic carbon contained in wastewater for the optimized membranes. IC* (inorganic carbon) = TC − TOC.

Table 2 lists the concentration of inorganic ions and selected micropollutants in the wastewater sample and in the permeates of the two membranes. In general, the two membranes have a similar selectivity. The specific selectivity for ions and total organic carbon (TOC) of the two membranes is plotted in Figure 8. TOC is also reduced by about 85%. Both membranes present a high rejection of organic pollutants. The concentration of imidacloprid (IMI) and 1,2,3-benzotriazole (BZT) were become undetectable after the filtration, while the concentrations of carbamazepine (CBZ) and 5-methyl-1H-benzotriazole (MBZT) were reduced by more than 95%. The two membranes show a high ability to remove inorganic ions. The highest rejection was observed for divalent ions as Mg^2+^ and Ca^2+^, SO_4_^2−^, and carbonates, which are likely the most representative species of inorganic carbon (IC) in these samples. In general, the rejection of monovalent ions is lower than the rejection of divalent ions, which is not necessarily a disadvantage, as many applications do not require to remove these ions and the permeation can result in a reduced trans-membrane osmotic pressure. 

## 4. Conclusions

In this study, we presented a simple method for the fabrication of the 5 mol% Al_2_O_3_-doped silica NF membranes. A surfactant, namely CTAB, was added during the sol-gel synthesis of the membrane material to reduce the hydrolysis and condensation rate of the aluminum alkoxide precursor, thus obtaining a homogeneous amorphous oxide network, and to act as pore-forming agent (template) during the calcination step. NF membranes were obtained by a single coating by dipping commercial alumina support in the Al_2_O_3_-doped silica sols. This approach may provide good bases for further development and fabrication of other metal oxide-doped silica membranes. 

We were able to reduce the thickness of the membranes down to 96 nm. However, membrane thickness, as observed at SEM, had a slight impact on the water permeability of our membranes, probably due to the penetration of the polymeric silica sol into the pores of the membrane support. The increase of the CTAB concentration in the coating sol has little impact on the pore size of the consolidated material, and therefore on the selectivity of the membrane layer. On the contrary, membrane permeability was greatly enhanced by the optimization of the surfactant concentration. During the filtration tests, the highest water permeability (2.3 L·m^−2^·h^−1^·bar^−1^) was achieved by the membranes prepared from the sol with S/O = 2.0, while the highest rejection values were obtained for the membrane coated from the sol with S/O = 0.50. All the membranes presented in this work have rather high rejection towards caffeine. 

Filtration of an effluent from a wastewater treatment plant indicates that the optimized membranes are capable of removing water-hardness ions such as Mg^2+^ and Ca^2+^, micropollutants and colored organic matter from real water systems. Thus, the membranes developed in the present study have high potential to be applied in tertiary treatments of wastewater or in the treatment of brackish water.

## Figures and Tables

**Table 1:** Fabrication parameters of the membrane samples synthesized in this work.

S/O ^#^	Reaction Time (h)	TEOS:Ethanol (mol:mol)	Al_2_O_3_:SiO_2_ (mol%)	Al_2_O_3_ + SiO_2_ Concentration ^§^ (g·L^−1^)
Membrane samples with different sol dilutions				
0.25	15	1:4	5%	11.8
0.25	15	1:4	5%	8.7
0.25	10	1:4	5%	6.5
Membrane samples with different CTAB concentrations				
0.25	10	1:4	5%	6.5
0.5	10	1:4	5%	6.5
1	10	1:8 *	5%	6.5
2	20	1:12 *	5%	6.5
4	40	1:32 *	5%	6.5

^#^ S/O = surfactant/oxide ratio = CTAB/(SiO_2_ + Al_2_O_3_) (mol/mol); * additional ethanol was added to dissolve the CTAB; ^§^ Sols were diluted before coating to achieve the oxide (SiO_2_ + Al_2_O_3_) concentration reported in this table.

**Table 2:** Concentration of ions and micropollutants in the wastewater sample as received and after the filtration over the membranes prepared from sols with S/O = 0.50 (Permeate 0.5) and S/O = 2.0. (Permeate 2). The data for imidacloprid (IMI), carbamazepine (CBZ), 1,2,3-benzotriazole (BZT), and 5-methyl-1H-benzotriazole (MBZT) take into account a preconcentration factor of 200 on solid phase extraction (SPE).

	Wastewater Treatment Plan Effluent (ppm)	Permeate S/O = 0.5 (ppm)	Permeate S/O = 2 (ppm)
Cl^−^	92.1	67.5	56.5
NO_3_^−^	20.2	12.0	14.4
SO_4_^2−^	40.9	15.7	13.0
Na^+^	103	16.0	14.4
K^+^	27.2	2.95	3.53
Mg^2+^	7.1	0.11	0.11
Ca^2+^	92	0.77	0.82
TOC (total organic carbon)	8.51	1.26	1.31
TC (total carbon)	69.0	6.59	6.97
IMI	3 × 10^−5^	-	-
CBZ	8.4 × 10^−4^	2.3 × 10^−5^	3.4 × 10^−5^
BZT	1.7 × 10^−4^	-	-
MBZT	1.7 × 10^−3^	1.5 × 10^−5^	2.0 × 10^−5^

## Supplementary Materials

The following are available online at https://www.mdpi.com/2079-4991/9/10/1368/s1, Table S1: MS-MS conditions of organic pollutants detected in the sample (DP-declustering potential, EP extensions potential, CE collision energy).
