# Supplementary material for: Surfactant-Assisted Fabrication of Alumina-Doped Amorphous Silica Nanofiltration Membranes with Enhanced Water Purification Performances
Source: Nanomaterials (Basel). 2019 Sep 24;9(10):1368. doi: 10.3390/nano9101368 (PMC6836250; doi:10.3390/nano9101368)
Supplement: Supplementary file 1 [file nanomaterials-09-01368-s001.pdf]

Surfactant-Assisted Fabrication of Alumina-Doped Amorphous Silica Nanofiltration Membranes with Enhanced Water Purification Performances

Xianzheng Ma [1], Katarzyna Janowska [1], Vittorio Boffa [1,*], Debora Fabbri [2], Giuliana Magnacca [2,3], Paola Calza [2] and Yuanzheng Yue [1]

[1] Department of Chemistry and Bioscience, Aalborg University, Fredrik Bajers vej 7H, 9220 Aalborg, Denmark; xm@bio.aau.dk (X.M.); kaj@bio.aau.dk (K.J.); yy@bio.aau.dk (Y.Y.)
[2] Department of Chemistry, University of Turin, Via P. Giuria 5/7, 10125 Torino, Italy; debora.fabbri@unito.it (D.F.); giuliana.magnacca@unito.it (G.M.); paola.calza@unito.it (P.C.)
[3] NIS Interdepartmental Center, Universitá di Torino, Via P. Giuria 7, 10125 Torino, Italy
* Correspondence: vb@bio.aau.dk; Tel.: +45-9940-3579

1. Analysis of inorganic and organic components in permeate and wastewater

1.1. Inorganic ions

A Thermo Scientific™ Dionex™ ED40 instrument equipped with a conductivity detector was used to quantify the concentration of anions in the permeates and in the effluent samples. Anions were analyzed using AS9HC column in the presence of 9 mM K_2CO_3 eluent at a flow rate of 1 mL min^{-1}. The concentration of cations was measured by a PerkinElmer® Optima 7000 DV ICP-Optical Emission Spectrometer equipped with WinLab™ 32 for ICP Version 4.0 software. Argon was used as the ICP torch gas with the Optima 7000 DV.

1.2. Organic components

Total organic carbon (TOC) was measured with a Shimadzu TOC-V CSH analyzer (catalytic oxidation on Pt at 680°C) equipped with an ASI-V autosampler, using potassium phthalate standards.

1.2.1 Sample preparation

200 ml samples of both permeates and effluent were prepared for solid phase extraction (SPE). Before sample elution the SPE cartridges Supelclean™ ENVI – 18 SPE (500 mg) were preconditioned by 4 ml of methanol and then equilibrated with 2 x 4 ml of mili-Q water. Then without allowing the column to become dry, the sample was introduced directly to the column, by means of a 0.45 μm PTFE tube, at a flow rate of approximately 5 ml/min. Afterward, cartridges were respectively eluted with 3 x 4 ml of ethyl acetate. The column was soaked in ethyl acetate for 5 min before the samples were allowed to run through the column. Then, elute was evaporated under a gentle nitrogen stream to volume 1 ml. The samples were stored in the freezer until analysis of CECs was performed.

1.2.2 Micropollutant analysis

In order to test the performances of membranes towards the organic pollutants, we analyzed the wastewater effluent and the permeate for the presence of some contaminants of emerging concern and in particular imidacloprid (IMI), ciprofloxacin (CPX), carbamazepine (CBZ), 1,2,3-benzotriazole (BZT) 5-methyl-1H-benzotriazole (MBZT). Analyses of these micropollutants in wastewater effluent and in permeate were performed by UFLC-SHIMADZU combined with QTRAP LC-MS/MS 3200 from SCIEX (Framingham, MA, USA). The analytes were separated on a Synergi 4μm Fusion RP (Phenomex, 50 x 2 mm) using a mobile phase

of water containing 0.05% (v/v) formic acid (eluent A) and acetonitrile (eluent B). The gradient elution was as follows: 0–10 min 5% to 100% B, 10-12 min 100% B, 12-12.5 min 100 to 10 % B, 12.5-14 min 5% B. The column oven temperature was set to 30 °C. An injection volume of 10 µl was used for all samples. Electrospray ionization (ESI) was applied in positive ion mode and multiple reaction monitoring (MRM) was used. ESI source parameters were as follows: ionization voltage was set at 2500 eV, ion source temperature was set to 400 °C, with curtain gas at 30 psi and collision gas set to medium; ion spray gas 1 and 2 was set at 60 psi and 70 psi respectively. Quantitative analysis was carried out using Analyst 1.6.3 software (SCIEX, Montreal, Canada), employing a calibration curve of pollutants standard prepared in ethyl acetate solvent. The most abundant MRM transitions of each target compounds and the optimized experimental parameters were listed in Table S1.

Table S1. MS-MS conditions of organic pollutants detected in the sample (DP declustering potential, EP extensions potential, CE collision energy).

Compounds	Quantification/Confirmation	Q1	Q3	DP	EP	CE
Imidacloprid _1	Quantification	256.2	209.2	37	5	21
Imidacloprid _2	Confirmation	256.2	175.4	37	5	22
Ciprofloxacin _1	Quantification	332.3	231.3	55	7	40
Ciprofloxacin _2	Confirmation	332.3	288.1	55	7	25
Carbamazepine _1	Quantification	237.3	194.2	36	7	21
Carbamazepine _2	Confirmation	237.3	179.3	36	7	43
1,2,3-benzotriazole _1	Quantification	120.1	65.1	48	7	31
1,2,3-benzotriazole _2	Confirmation	120.1	92.2	48	7	24
5-methyl-1h-benzotriazole _1	Quantification	134.2	77.1	44	7	34
5-methyl-1h-benzotriazole _2	Confirmation	134.2	79.4	44	7	26
